# Challenges and improvement needs in the care of patients with central diabetes insipidus

**DOI:** 10.1186/s13023-022-02191-2

**Published:** 2022-02-16

**Authors:** H. Teare, J. Argente, M. Dattani, J. Leger, M. Maghnie, M. Sherlock, G.-C. Ali, J. Francombe, S. Marjanovic

**Affiliations:** 1 RAND Europe, Westbrook Centre Milton Road, Cambridge, CB4 1YG UK; 2 Department of Pediatrics and Pediatric Endocrinology, Hospital Infantil Universitario Niño Jesús, Instituto de Investigación La Princesa, Universidad Autónoma de Madrid, Madrid, Spain; 3 Centro de Investigación Biomédica en Red (CIBER) de Fisiopatología de la Obesidad y Nutriciόn (CIBEROBN), Instituto de Salud Carlos III, Madrid, Spain; 4 IMDEA, Food Institute, CEIUAM+CSI, Madrid, Spain; 5 UCL Great Ormond Street (GOS) Institute of Child Health, University College London, London, UK; 6 Great Ormond Street Hospital for Children, London, UK; 7 Assistance Publique-Hôptaux de Paris, Pediatric Endocrinology-Diabetology Department, Reference Center for Growth and Development Endocrine Diseases, Robert Debré University Hospital, Université de Paris, NeuroDiderot INSERM UMR 1141, 75019 Paris, France; 8 Department of Pediatrics, IRCCS Istituto Giannina Gaslini, Genoa, Italy; 9 Department of Neuroscience, Rehabilitation, Ophthalmology, Genetics, Maternal and Child Health, University of Genova, Genoa, Italy; 10 Department of Endocrinology, Beaumont Hospital and Royal College of Surgeons in Ireland, Dublin, Ireland

**Keywords:** Central diabetes insipidus, Disease burden, Healthcare improvement, Healthcare systems, Rare disease, DDAVP

## Abstract

Central diabetes insipidus (CDI) is a rare condition, with significant impact on patient health and well-being. It is a chronic condition which usually requires meticulous long-term care. It can affect both children and adults. There is limited literature considering the needs and challenges inherent in providing high quality care to patients with CDI, across the care pathway. This paper seeks to address this gap by providing a unique and well-rounded understanding of clinical and healthcare systems-related challenges. It draws on insights from the literature, from direct clinical experience contributed by five clinicians as co-authors (providing insights from France, Ireland, Italy, Spain and the United Kingdom), and from patient perspectives provided through interviews with patient representatives from three patient organisations. We identify clinical challenges related to the diagnosis of CDI, including differentiating between other similar conditions and determining the underlying aetiology. Treatment is challenging, given the need to tailor medication to each patient’s needs and ongoing management is required to ensure that patients continue to respond adequately to treatment. Ongoing support is required when patients switch between formulations. We also identify healthcare systems challenges related to limited awareness of CDI amongst primary care physicians and general paediatricians, and the need for highly skilled specialist care and appropriate workforce capacity. There is also a significant need for raising awareness and for the education of both healthcare professionals and patients about different aspects of CDI, with the aim of supporting improved care and effective patient engagement with healthcare professionals. We reflect on this information and highlight improvement opportunities. These relate to developing guidance to support patients, carers, primary care physicians and general paediatricians to identify clinical features earlier, and to consider CDI as a possible diagnosis when a patient presents with suggestive symptoms.

**Supplementary Information:**

The online version contains supplementary material available at 10.1186/s13023-022-02191-2.

## Central diabetes insipidus: a rare disease with significant impacts on patient health and well-being and a limited evidence base on how to improve patient care

Central diabetes insipidus (CDI) is a rare disease affecting approximately 1 in 25,000 individuals [4]. It has significant impact on patient health and well-being and usually requires long term care. The evidence base on how to care for patients with this condition is fragmented. There is a need to advance knowledge on the diversity of challenges to patient care and to identify scope for improvement.

In this paper, we provide an overview of the diagnosis, treatment and management of patients with CDI to consider the diversity of factors that play a role in determining the quality of patient care. We identify challenges and improvement needs in both adult and paediatric populations. We draw on insights from a narrative literature review, complemented with the experiential knowledge of clinical experts and the views of representatives from patient associations.

CDI is characterised by hypotonic polyuria and polydipsia resulting from decreased concentrations of arginine vasopressin (AVP), also known as antidiuretic hormone (ADH) [4, 11]. Other symptoms can include fatigue, dizziness, hypotension and tachycardia related to dehydration, and hypernatremia which is a direct consequence of CDI [15, 24]. Severe dehydration is a particular challenge in young children, who may not as yet be able to self-regulate thirst. Children can also experience vomiting, constipation, fever, irritability, sleep disturbance, retardation of growth, failure to thrive and potentially developmental disability caused by repeated dehydration and hypernatremia [9, 15, 18, 20].

The disease is caused by damage to AVP-producing magnocellular neurons in the hypothalamus. The degree of deficiency of AVP is the main factor that determines the severity of CDI symptoms. Damage to this region of the brain can occur either due to traumatic injury [3, 11] or non-traumatically, for example in response to a tumour, infiltration, infection or inflammation, or less frequently as a result of genetic mutations [1, 21]. Hereditary forms of CDI present early in life, whereas acquired forms can occur at any age. CDI has a similar prevalence in men and women. A patient’s quality of life is often impeded as a result of the condition, for example from sleep being disturbed by nocturia [25]. There is also a risk of fatality in the most severe cases, which can happen because of chronic or severe dehydration, hypernatremia, fever and cardiovascular failure due to a compromised ability to regulate blood pressure [5, 7]. Adipsia can increase risk of death [12]. In patients with CDI, an intact thirst mechanism is critical for protecting against hypernatremia and dehydration.

CDI is one of four conditions associated with polyuria and polydipsia, along with nephrogenic diabetes insipidus (NDI), primary polydipsia and gestational diabetes insipidus [7]. Once diagnosed, treatment of CDI is generally effective, with desmopressin (DDAVP, D-amino D-arginine vasopressin- an AVP analogue) used as the mainstream treatment to help regulate fluid in the body. However, it can be challenging to identify and accurately diagnose patients and to get them onto appropriate treatment [7, 8, 14, 15, 20, 23, 24]. Optimising dosages and treatment formulations and ensuring personalised care is also not straightforward [1, 22, 23]. In addition, it can be challenging to ensure that patients remain stable on treatment over time, including when managing CDI alongside other concurrent conditions, particularly adrenocorticotropic hormone (ACTH) deficiency. Furthermore, the lifelong treatment that patients with CDI often require [7] places significant demand on healthcare systems for ongoing management and monitoring.

The aim of this review is to expand on the key features of the care pathway and the associated factors and challenges that influence patient care, and to reflect on areas in need of improvement.

## Methods

The paper combines findings from a narrative literature review, insights based on experiential knowledge from five leading clinical experts from the United Kingdom (UK), Ireland, Italy, Spain and France who are also co-authors of the paper, and the views of patient representatives from three associations active in the CDI area (the Pituitary Foundation, UK; the Pituitary Foundation, Ireland; and the Italian Family Association of Septo-optic Dysplasia and Pituitary Abnormalities, Italy).

### Narrative review

The narrative literature review was conducted using a systematic search strategy, with the search conducted in October 2020. It included papers published between October 2015 and October 2020, with specified criteria for prioritising papers which were then subjected to thematic analysis. It focused on understanding the care pathway for patients with CDI, challenges and wider influences on patient care, and improvement needs. A total of 25 papers were included in the review; further detail of their selection is included in the Additional file 1.

### Focused discussion with clinical experts

To refine, nuance and build on insights from the literature, the paper co-authors—representing healthcare services research experts from the not-for-profit research institute RAND Europe (SM, HT, JF, DRR, GCA) and clinicians working with patients with CDI in France (JL), Ireland (MS), Italy (MM), Spain (JA) and UK (MD)—engaged in focused discussion. This included individual discussions between a RAND Europe health services researcher (either SM, HT, DRR, or JF) and each individual clinician, a total of five one-hour discussions. It also included group dialogue through two online workshops bringing together the clinical experts and RAND Europe researchers (workshops were held on 23 and 27 November 2020). The workshops were opportunities for collective discussion about care-related improvement needs. They also served to explore healthcare system related influences on quality of care and access to care in more depth, given that these aspects are under-explored in the literature. Clinical co-authors from four countries also gathered estimations of the costs of care for a patient in their local healthcare setting (i.e. hospital) to provide a high-level indication of the estimated costs related to hospital care that are incurred during the patient pathway. Although these are just estimates, and do not cover primary care costs for example, they provide initial insights on the resource implications of caring for patients with CDI which can be built on in future studies, and that extend beyond medicine costs alone.

### Interviews with patient representatives

Perspectives were gathered from three patient representatives from associations which support patients with CDI in Ireland, Italy and the UK (unfortunately this was not feasible in Spain or France). Their perspectives helped to better understand how patients experience CDI and their views on future priorities to improve the provision of care. Patient representative views are referenced in brackets with PR-INT X, with X being the code number for an individual interviewee. In some instances, where there is a risk of identity disclosure or linkage of information to a country context, or in light of informed consent, we withhold a reference to preserve anonymity.

We have thematically cross-analysed and triangulated insights from these various sources to develop a multifaceted understanding of care pathways, associated challenges and improvement needs.

There are some caveats to consider when interpreting our findings. The literature review covered high income countries and additional insights from low- and middle-income countries would help to enrich these findings. The workshops and one-to-one discussions with clinical experts are limited to the countries they work in and are based on their personal experience—however these are all leading experts in their country contexts. The interviews are with patient representatives of a limited number of patient organisations who could engage with the study, in part related to the small number of existing patient organisations active in CDI. The cost estimations in particular are limited by the availability of data and detail that could be provided, and thus can only provide an initial indication of cost rather than a comprehensive overview. Despite these caveats, the insights from the literature review and complementary views from clinical experts and patient representatives provide a valuable contribution to enriching our understanding of how care for CDI is provided and what the challenges and improvement needs are.

## The care pathway and associated challenges related to diagnosis, treatment and ongoing management

### Diagnosing CDI

In children and adults, CDI is diagnosed using a series of tests to confirm polyuria and polydipsia and to try to understand the underlying cause. Investigations tend to follow a logical progression of clinical history and examination followed by biochemical and endocrinological assessment, followed by radiology. The approach to diagnosis is similar across the different healthcare settings considered in this paper [workshop insights]. In countries where primary care is generally the first point of access to healthcare, patients with CDI will usually first present to a primary care physician (or general practitioner) having experienced symptoms such as dehydration, thirst and frequent urination [PR-INT1, PR-INT2, PR-INT3]. Following basic tests by a primary care physician, in most cases patients will be referred to a specialist endocrinologist for further diagnostic testing [PR-INT1, PR-INT2, PR-INT3].

Both adults and children follow a similar diagnostic pathway. A water deprivation test (WDT), during which the patient is not allowed to drink, has been the gold standard for diagnosing CDI [20]. It involves meticulous and regular measurements of urine production, blood electrolyte concentrations, plasma and urine osmolality and weight for approximately 7 h (and occasionally for shorter durations in children, if cessation of the test is necessary due to weight loss or hypernatremia) [20]. This usually takes place in a specialist hospital setting supported by an endocrinologist and specialist nurses, as water deprivation requires careful monitoring [24]. Patients can find the WDT test extremely unpleasant and challenging [PR-INT2, PR-INT3]. The water deprivation test is sometimes done in modified versions and for shorter periods of water deprivation in some paediatric patients and may be avoided in infancy if hypernatremia with low urine osmolality is present. Parents of children with CDI symptoms may also be asked to measure their child’s fluid balance [PR-INT3]. A WDT may not always be necessary for pregnant women, for whom such a test could be overly cumbersome [22]. There is potential for increased use of alternative diagnostic tests in the future [PR-INT2], for example measurement of copeptin and the use of dynamic tests such as arginine or hypertonic saline infusions. However, the current use of such tests across different countries remains unclear.

Magnetic Resonance Imaging (MRI) is essential to determine potential causes of CDI—for example it can show thickening of the pituitary stalk which might indicate inflammation/autoimmune conditions, tumours or other disorders, or a neoplastic process in the sellar region [7]. However, there are challenges associated with using MRI to identify etiological markers of CDI [20]. Absent neurohypophysis can be diagnosed by MRI if the posterior pituitary bright spot is missing.

Differentiating between the various conditions which share symptoms of polyuria and polydipsia can be challenging and can take considerable time [7, 8, 12]. Existing diagnostic tests can risk false diagnoses, both positive and negative, particularly in patients with mild or partial forms of CDI [23 PR-INT2]. Understanding the potential cause of symptoms can help in the diagnostic process, as some events, such as traumatic brain injury are associated with increased likelihood of CDI and can, together with other tests, help to confirm whether a patient has CDI or another condition. Therefore, a full patient history is an important part of the diagnostic process [11].

Timely diagnosis is impeded if symptoms are not noticed promptly [10], or if other aetiologies for polyuria and polydipsia are investigated first [13]. A lack of awareness among primary care physicians regarding the symptoms of CDI [PR-INT1, PR-INT2] can also impede timely diagnosis and referral. The time to diagnosis can vary across countries.

Costs associated with diagnosis can vary a great deal depending on the speed of diagnosis and the complexity of the underlying aetiology, and if additional support is needed, for example intensive care support in instances of traumatic brain injury, or genetic testing to confirm cause (see the cost section below).

### Treating CDI: treatment initiation and optimisation

Once a diagnosis has been reached, it is necessary to tailor treatment to the individual; given the marked variability in patient response to treatment, each patient needs a personalised protocol [2, 9, 20].

As introduced previously, CDI is most often treated by replacing AVP with a synthetic vasopressin analogue called desmopressin, in both adult and paediatric populations [1, 6, 8, 10 PR-INT1, PR-INT2, PR-INT3, workshop insights]. In all patients with chronic CDI, a common starting point for treatment is to first address nocturia, by administering the first dose of desmopressin before bed [22]. The patient's response and dose will be measured and adjusted over time until their symptoms are effectively controlled. Initial treatment optimisation generally involves starting with a low dose and gradually increasing if needed [10]. Some adult patients may only need a single dose per day to control their symptoms overnight (although the strength of the single dose can vary significantly between patients), while severe cases might need up to 200 μg twice or three times a day (occasionally up to 1 mg in total) [11].

Infants and young children will require much lower doses than adults, which will need to be adjusted over time, as the children grow and develop. Young infants are also dependent on fluid such as breast milk or formula for nutrition, and this can present additional challenges to fluid regulation and dose optimisation. This is because the primary therapeutic goals of treating CDI in young children are to reduce polyuria and decrease excessive thirst to support appropriate levels of fluid uptake, and to ensure appropriate growth. Children require adequate fluid intake and various doses of DDAVP ranging from a low dose of 10 µg/day for infants in cases of neonatal DI to 60 µg/day three times a day during later childhood, although higher doses could be necessary for individualised patients. Careful exact dosing by cutting the available oral disintegrating tablet DDAVP formulation should be performed, particularly for young paediatric patients. Starting with low doses and titration of the dose is important.

Given desmopressin’s mode of action, ongoing patient monitoring during treatment dose optimisation is important. This is because of the risk of developing hyponatremia if too little water is excreted or too much fluid ingested [13], or hypernatremia [8, 12, 16, 17] if too much water is lost or too little fluid ingested. It is important to allow water offload in this process, to prevent hyponatraemia. Optimisation therefore involves a specialist endocrinology clinic to determine patient response to treatment and whether the dose needs to be further adjusted. It usually takes 2–3 days, or up to a week as an inpatient, but can take longer for infants [workshop insights]. Treatment optimisation occurs during the same admission as the diagnostic stage.

Desmopressin is available in several different formulations. Decisions relating to which formulation is most appropriate depend on the patient being treated. It can be administered orally as a tablet or as an oral disintegrating tablet (ODT), as a buccal preparation, as a nasal spray, or parenterally (usually intramuscularly or intravenously). The latter may be required in patients that are required to fast pre-operatively or for patients needing intensive care [1, 6, 8, 10, 24, PR-INT1, PR-INT2].

Desmopressin lyophilizate sublingual tablets are a valuable option for treating CDI in infants and young children, with evidence of more stable absorption than intranasal formulations and oral tablets [9, PR-INT1 and PR-INT2]. However, it can be difficult to split these sublingual tablets into small enough doses for infants. Subcutaneous DDAVP administration enables the administration of small doses to infants but this can lead to more variable sodium concentrations than other formulations [9, and the experience of an expert involved with this research suggests that subcutaneous DDAVP should only be administered in intensive care or post-operatively. According to one patient representative and in their country context, paediatric patients are generally initiated on a nasal spray due to ease of administration, but the formulation may be changed depending on the patient’s response [interview reference withheld to preserve anonymity]. In mild cases of CDI in some countries, clinicians may consider not prescribing DDAVP and instead suggest strategies for managing fluid balance and using fluid replacement [interview reference withheld to preserve anonymity]. Different formulations also take different times to act and hence some patients may prefer formulations which provide relief quicker [PR-INT2].

Treating CDI with desmopressin is generally safe and effective and shown to improve patient quality of life [11], but can come with side effects. Side effects, while rare, vary slightly between formulation. The intranasal spray, for example, may lead to dizziness, eye irritation, headache, flushing, nausea, vomiting, rhinitis or epistaxis and tachycardia. Evidence suggests that oral DDAVP is better tolerated by some patients [20]. Dose adjustment can help to mitigate side effects [workshop insights].

Although desmopressin is the main treatment for CDI across different types of patient groups at present, some other treatments are used in some countries (even if not specific for use in CDI) with slightly different modes of action and side effects. These include thiazide diuretics, carbamazepine, chlorpropamide, clofibrate and indapamide [9, 10, 13, 15]. However, desmopressin remains by far the most commonly used form of treatment [10].

There is a lack of data on the costs associated with treatment optimisation and these costs are likely, at least in part, to depend on the complexity and length of the optimisation process.

### Managing patients with CDI over time

Given that CDI is usually a chronic condition, lifelong management and ongoing monitoring are necessary to ensure that patients respond well to their specific treatment over time. Ongoing care is also necessary to ensure that any changes to treatment are carefully managed [7]. It is difficult to predict how a patient will respond to treatment. Patient response can be influenced by their ability to adhere to treatment regimens, for example to control fluid intake or regularly take medication. It can also be influenced by other factors such as other illnesses—for example a cold which might affect how well a patient absorbs desmopressin nasal spray, or vomiting which will influence the intake of an oral tablet [2, 11, 15, 16]. Patient response to a specific treatment dose and formulation can also be influenced by lifestyle factors such as foreign travel to a warm country due to changing water intake behaviours or participating in sport which might impact fluid requirements [workshop insights]. Annual or biannual clinic appointments with an endocrinologist are often required to ensure treatment doses and formulations remain effective. Such clinic appointments are the main healthcare service-related cost associated with the longer-term management of CDI, as outlined in the cost section below.

As part of patient management, it is sometimes necessary to not only adapt dosage but also to switch between different formulations of desmopressin. This can happen if patients find a specific formulation easier to administer [15] or due to potential supply issues, or differences in the effectiveness of a formulation [two interviewees, interview references withheld to preserve anonymity]. Switching between formulations requires dose titration to optimise the dose for each patient, as it is not possible to predict how patients will respond to new formulations based on their previous treatment protocols [14, 15]. This is related to the absence of conversion factors between different formulations. Switching between formulations will require further in-clinic support, which can add to the cost of treatment. Establishing conversion factors between different formulations is challenged by the fact that different formulations have different bioavailability and that there is diversity in how individuals respond to any specific formulation and dose. The UK electronic medicines compendium^1^ provides a correlation table for oral tablet and melt formulations for adults, but this is based on bioavailability correlation research which tends to take place in healthy volunteers, rather than patients with CDI. Whereas such information may help orient clinicians, it cannot guide clinical decision-making given the highly personalised nature of dose optimisation for CDI, and the frequent need for starting with lower doses as part of the dose optimisation process.

Specialists—generally endocrinologists—determine which brand (or generic) and formulation to use, depending on what they think is best for the patient or what they are most familiar with [workshop insights]. In the countries included in this research, the use of branded versions appears more common at the point of prescription, possibly because generic versions are not available or because of clinician preference [workshop insights]. Whether there is therapeutic equivalence between branded or generic products for CDI has not been researched. If patients are followed up in primary care, primary care physicians can make decisions to switch between branded or generic versions, if generic versions are available. However, they generally would not change the dose for paediatric patients without discussion with a specialist. In some countries, repeat prescriptions tend to be organised by primary care without specialist involvement, although the specialist centre would still direct dosage and monitor the patient as needed.

Long term management decisions other than those related to medication will also be dependent on whether patients are able to regulate thirst themselves, or whether they are adipsic. Patients who are able to experience thirst are generally encouraged to avoid excess fluid intake, and drink to thirst rather than habit. For adipsic patients, it is more difficult to keep track of how much water intake is needed [11]. Therefore, a routine approach to drinking with a daily fluid regimen based on strictly regulated fluid intake with fixed amounts of water, is important to help mitigate the risk of hyper- or hyponatremia [9]. This can be particularly challenging if patients contract other illnesses, for example if they are vomiting or eating less or have diminished consciousness [11]. Fasting for a surgical procedure can also introduce risk, and must be supervised by a specialist, particularly if it requires a change in treatment formulation, for example if the patient usually administers ODT [11]. According to experts consulted for this research, physicians should be aware of associated, treatable hypothalamic abnormalities such as obesity, sleep apnoea, seizures and thermoregulatory disorders when managing patients with adipsic diabetes insipidus. From a fluid balance perspective, these patients require regular DDAVP to treat their CDI but also need a daily fluid prescription in order to maintain euvolaemia (an appropriate blood fluid volume within the body) and eunatraemia (an appropriate concentration of sodium in blood plasma). Adjusting the desmopressin dosage and fluid intake for adipsic patients is generally done in a hospital setting and regular weighing and checking of serum sodium levels is necessary.

Similarly, patients with CDI diagnosis prior to pregnancy who are already receiving doses of exogenous vasopressin or desmopressin may require increased doses during pregnancy and additional monitoring to ensure that there are no complications due to increased dosages [1].

Given the impact of CDI on growth and development in infants, more frequent monitoring is also particularly important in this vulnerable patient population. This will include monitoring serum sodium, weight and hydration to allow doses to be modified, and home-monitoring including weighing wet ‘diapers’ [25]. According to an expert involved with this research, daily weighing of an infant/young child patient should make it possible to detect abnormal weight gain, and plasma sodium concentration should be monitored frequently to reduce the risk of hyponatremia. The challenges associated with dose adjustment mean that it is important to involve the relevant experts early in this process to support any required changes to dose [10]. In paediatric inpatients who are unable to tolerate oral desmopressin (for example while they are unwell with other conditions such as gastro-intestinal issues) a switch to parenteral therapy may be required, although such cases are rare. This would typically be delivered intramuscularly, but intravenous pitressin infusion (vasopressin injection) can also be used. If this is different from the patient’s usual desmopressin formulation, particularly close monitoring is required as it is not possible to predict how the patient will respond [10].

Both for adults and children, management of the condition at home is also an important part of the patient and/or carer journey [PR-INT1, PR-INT3]. Although patients will tend to have regular six-monthly or annual follow-up appointments, ensuring appropriate medication adherence and administration at home is key [PR-INT2]. Lifestyle issues also require patients to engage with ongoing management or monitoring through their own behaviours. For example, stress, menstruation or unusual consumption of a lot of salty/sweet foods may require desmopressin to be taken at specific times in the day [PR-INT2].

## Influences on patient care and the care pathway within healthcare systems

The diagnosis of patients with CDI, decisions about treatment dosage and formulation, and ongoing patient monitoring and management are strongly influenced by features of the wider healthcare system in which patients are treated, as discussed below [4, 5, 7–11, 13, 16, 20–23, 25].

### Influences related to skills and workforce capacity

Supporting patients with CDI requires specialist skills involving endocrinologists and including paediatricians and nurses to support infants and children. Patient support will also draw on other specialties depending on the cause of CDI and specific patient needs [9]. For example, patients with CDI as a result of a tumour, or as a result of tumour surgery, will require oncologist and neurosurgeon involvement in care. Patients with CDI as a result of traumatic brain injury may need intensive care teams, and patients with CDI in pregnancy will need additional support, for example from obstetrics [workshop insights]. Appropriate support requires the healthcare system to have sufficient workforce capacity and a cadre of highly skilled staff across specialties [2, 11, 15, 16].

Several of the healthcare systems included in this paper have specialist centres that support patients across a large geographic region. This allows expertise to be concentrated but can also require patients to travel long distances to seek specialist advice and support [PR-INT1, PR-INT3]. This places significant emphasis on the first stages of patient support, often through primary care or other departments such as emergency medicine or oncology, to recognise specific patient needs [workshop insights]. In some countries, the limited number of hospitals with relevant clinical expertise can also create long waiting times for treatment [PR-INT1] or necessitate remote management.

Healthcare professionals in primary care (both primary care physicians and nurses) can sometimes lack sufficient awareness about the symptoms of CDI which can complicate timely diagnosis and referrals [PR-INT1, PR-INT2, PR-INT3]. This can sometimes lead to inappropriate treatment [PR- INT1, PR-INT2]. Primary care physicians are also sometimes not appropriately trained on the importance of carefully managing and monitoring appropriate treatment dosing and formulations as patient conditions change [workshop insights]. In a hospital care setting, not all physicians always understand how desmopressin acts, which can complicate the inpatient care of patients with CDI who are admitted for other conditions [8, 25].

Adjusting treatment formulations and dosages over time also requires significant skills to make appropriate decisions and to support personalised care, given that different formulations have significantly different bioavailability and that patient response differs [16, 19]. Any potential changes in relation to bioavailability between branded and generic products require further research (as there are gaps in evidence on this issue), to inform policies on generic substitution and automatic dispensing.

The COVID-19 pandemic has also created specific challenges for patients with CDI. These include limited access to primary care physicians to get a referral to an endocrinologist [interview reference withheld to preserve anonymity], challenges accessing and undertaking diagnostic testing [PR-INT1], and lack of sufficient follow up with clinicians related to ongoing management needs. According to one patient representative, some patient support groups and helplines saw an increase in enquiries as a result [interview reference withheld to preserve anonymity]. There is a need to consider how patients can best be cared for in the context of any future pandemics, both in terms of remote care and any essential face to face contact with clinicians.

### Influences related to patient engagement with care related decisions and behaviours

Patients and/or caregivers have an important role to play in the ongoing management of CDI and in engaging with healthcare professionals about optimising treatment [17]. For example, patients may have preferences for specific formulations [workshop insights] in light of their absorption, the types of complications experienced or how straightforward the formulation is to administer. Patient engagement is also essential in the context of monitoring treatment effectiveness and reporting any changes in effectiveness to healthcare professionals [6, 17], as well as in the context of ensuring dose adherence and compliance with treatment [8, 20]. For some formulations, patient skills in administering the treatment, such as dexterity in administering intranasal formulations, play a role in treatment effectiveness [16].

Patient education can help equip patients with knowledge about how to manage their disease, what to look out for and side effects [8, 12, 23], which can in turn support effective communications with primary care physicians about treatment and monitoring needs [workshop insights]. In the UK, the Pituitary Foundation provides resources that patients can share with general practitioners if they suspect they have CDI. There are also efforts in some countries to rename the condition so as to remove the term ‘diabetes’ to avoid confusion with diabetes mellitus [interview reference withheld to preserve anonymity].

While there is a need for information and awareness raising regarding the symptoms of CDI [PR-INT1], there is also a need for more education on how families can support treatment and management. This includes a need for improved access to information about water intake, food, salt levels and measuring water balance [PR-INT3]. Beyond patients, interviewees also highlighted the need for better public understanding of CDI [PR-INT1, PR-INT2], for example to avoid patients being refused access to toilet facilities in some settings like shops [PR-INT2]. Awareness raising amongst other professions such as teachers, university lecturers and air stewards may also be needed so that patients can manage their condition with as much ease as possible [PR-INT2].

Those patients who are on both glucocorticoid (for example in the case of panhypopituitarism) and DDAVP replacements may need educational support about specific sick day management. Glucocorticoids are essential for the excretion of water. Some patients who have central diabetes insipidus also have anterior pituitary dysfunction and as such require glucocorticoid replacement. When a child taking both regular glucocorticoid and DDAVP becomes unwell, the parents may need to ensure that the glucocorticoid is doubled or trebled, and that the child is passing urine before giving further doses of DDAVP. In the event of inadequate glucocorticoid administration, water will not be effectively excreted and the administration of DDAVP can lead to water intoxication and hyponatraemia with related complications such as seizures.

### Availability and use of appropriate treatment formulations and medicines management and the role of regulation

The availability of appropriate formulations that are safe and effective as well as convenient to administer plays a role in treatment decisions and their appropriateness for users [12, 16, 20, 25]. Communication between clinicians and patients in relation to why some formulations may be available and why there may be shortages of others (or lack of availability) matters in terms of patient-centred care and good patient-physician communication [workshop insights]. Patient representatives raised issues regarding treatment availability and supply [interview reference withheld to preserve anonymity]. In one country, an interviewee commented that intranasal solution and nasal spray forms of desmopressin have been unavailable since the autumn of 2020, with patients and support groups not knowing when the supply will return [interview reference withheld to preserve anonymity]. Many patients using nasal formulations have subsequently transferred to sheets/melts, which created further supply issues.

Regulation plays an important role in ensuring the availability of appropriate treatments and in promoting good practice. For example, the intranasal spray formulation of desmopressin is no longer approved by the United States Food and Drug Administration, and this has led to a switch to the tablet or melt formulation in many countries [15]. Similarly, it is important that regulatory officials understand reasons for certain decisions, for example in relation to medicine switching and reasons for product recalls, to support national decisions and guidance that can impact patients directly [15].

While there is literature discussing good practice for specific patient groups (e.g. children and infants) [9], and guidance on inpatient management of CDI [4], we did not identify national level guidelines in the sample of papers reviewed relating to the countries of interest, namely: France, Ireland, Italy, Spain or the UK.

### Costs and effective resourcing to ensure appropriate capacity

Economic considerations will vary across healthcare systems, and economic burden may play a role for some patients [3, 25]. For example, a US-based study reported a one-month supply of intranasal DDAVP to cost USD 245.80 (based on 2016 data, equivalent to 179.43 euros in 2016), while a one-month supply of subcutaneous DDAVP cost USD 565.25 for treating infants in the USA (based on 2016 data, equivalent to 412.63 euros in 2016^2^) [25]. This can have significant cost implications over a patient’s lifetime. Data from the UK^3^ provides costs for desmopressin acetate, with 90 oral tablets of 100 μg individual doses reported to cost £44 (based on 2016 data, equivalent to 46.62 euros in 2016) and a pack of 100 sublingual disintegrating tables of 60 μg individual doses reported to cost £51 (based on 2016 data, equivalent to 54.03 euros in 2016). A pharmacoeconomic analysis of vazomirin spray (a treatment used in Russia with desmopressin as the active ingredient) compared with other forms of administration concluded that the cost of vazomirin spray, compared to vazomirin tablets is reduced by 45–48% in patients after resection of chiasmo-sellar region (CSR) tumours [3]. In general, cost considerations are an important factor in making treatment decisions. Although this research study did not focus on gathering specific medicines cost data in individual countries as part of its scope, the costs of CDI treatment are likely to vary across different geographies, as is the case with many medicines. Therefore, costs in a US context cannot be used to make inferences about costs of medicines in different European countries.

Clinical experts from four countries shared cost estimations for their own hospital settings (see Table 1). In all four countries, the costs of healthcare service provision (i.e. consultations and diagnostic tests) are covered by the public healthcare system. However, the clinical experts also noted that given the personalised nature of CDI treatment and management, costs per patient can vary substantially within any one given context [workshop insights]. This means that healthcare services costs are not directly comparable between countries as well as between different hospital settings. In terms of medicine costs, in health systems with a mixture of public and private funding, desmopressin is usually covered as part of a long-term illness scheme, and therefore publicly funded [workshop insights].

**Table 1.** Examples of costs for treating a patient with CDI—estimates from a sample of hospital settings (Country names withheld to preserve anonymity, all costs expressed in Euro equivalents)

Stage for each country	A (adults)	B (paediatric)	C (paediatric)	D (paediatric)
Diagnosis	The costs presented relate to the diagnosis and management of CDI as a result of acute CDI. This will differ from adults presenting with polydipsia and polyuria, and can take over six months longer to diagnose Initial visit including blood tests: EUR 750–800	Cost of hospitalisation and first diagnosis: EUR 1500–4500	Including admission in the paediatric endocrine unit for initial evaluation, diagnosis and treatment initiation (four days), and for repeated evaluation every six months during the first three years after diagnosis (two days for clinical, biological and cerebral MRI assessments and treatment optimisation): EUR 50,000	Cost estimates for paediatric patients including admission into the paediatric endocrine unit for initial evaluation, diagnosis and treatment initiation (three days), and for repeated evaluation every six months during the first three years after diagnosis: EUR 37,600 (costs of DDAVP not included)
	One day of testing including water deprivation test: EUR 2300–2500	In patients who need cerebrospinal fluid analysis and/or pituitary stalk biopsy, additional costs: EUR 2000–3000		
	MRI: EUR 400	Supporting patients (based on the imaging work-up) every six months for the first two years of treatment: EUR 400–1000		
	Neurosurgery including hospital stay if required: EUR 10,000–15,000 (approximately EUR 1500 per day)	Idiopathic CDI may have additional costs a long time after diagnosis as a result of complications developing – specific cost data not available.		
	ICU stay if required: EUR 10,000 per day	Genetic diagnosis may be needed in rare cases: EUR 500		
Long term follow-up	Outpatient visits: EUR 300 for two visits per year		One clinical and biological examination every six months: EUR 3000 per year (for following seven years post diagnosis)	Approximately EUR 2350 per year.
	Data on costs of treatments (therapeutics) is not available	Desmopressin: EUR 6000 for 10 years	Desmopressin treatment (on average 60 ug x 3/day): EUR 6500 for 10 years	Data on costs of treatments (therapeutics) is not available
			If other hormonal treatment is required, such as growth hormone treatment (with about 1 mg per day), an additional cost of up to EUR 90,000 for 10 years can be incurred	
Approximate total for 10 years	EUR 8450–34,200 (more if longer than one day in ICU, and not including medication costs)	EUR 12,100–28,500	Approximately EUR 60,000 (depending on patient complexity) (if growth hormone treatment if needed, additional costs are incurred)	Approximately EUR 54,000 (depending on patient complexity)

Given the limitations set out above, the information in the table below is shared purely for illustrative purposes and cannot be used to infer costs for an entire system nor for other settings. It seeks only to provide some initial information on what healthcare costs are like in some settings, given the paucity of any such insights in the existing literature. The data in the table can also not be aggregated into higher level categories to make inferences about overall diagnosis and care costs, given that different settings collect different types of cost-related information. However, the table begins to shed light on the potential cost items implied in the care of patients with CDI and indicates that the healthcare service costs are not negligible.

The data provided for each country refer to a specific patient population (paediatric or adult CDI patients), with particular mechanisms for data collection and provision based on information clinicians could gather. Other caveats to bear in mind are that the figures provided could be broken down by initial diagnosis, long term care and estimates of total costs for a 10-year timeframe and indicate highly variable ranges, in part dependent on patient complexity. For example, cost can be influenced by whether additional complex testing is needed beyond standard tests for CDI, such as additional cerebrospinal fluid analysis or biopsy to determine the cause of CDI, and by whether patients with CDI caused by traumatic brain injury need to be supported by neurosurgery. It can also be influenced by how long a patient needs to stay in hospital and whether they then need intensive care. If other hormonal treatment is required in addition to desmopressin, such as growth hormone treatment, there will be an additional cost. Therefore, costs of care are highly individualised depending on the specific circumstances of a patient. However, despite these figures being only indicative and context-specific and illustrating diverse cost-ranges across contexts, they indicate that the costs of caring for patients with CDI are not negligible and reinforce key insights we have gained about the complexity of the process.

It is worth noting that there can also be additional out of pocket costs for patients, such as the cost of items necessary for coping with the condition, for example ice cold water, nappies and toilet rolls [PR-INT2].

## Discussion and conclusion: towards improved care pathways

Within this paper we have considered current practice in the diagnosis, treatment and management of CDI, and discussed the associated challenges relating to differential diagnosis and highly personalised treatment and management.

We have shown that CDI requires complex patient support. This means care delivery needs to be flexible to meet individual patient needs. The fact that patient response to the mainstream treatment desmopressin can change over time makes long term management needs difficult to predict. This highlights the need for patients to be well-informed in order to effectively engage with healthcare professionals in primary and specialist care. It also highlights the need for highly skilled healthcare professionals to identify symptoms, determine optimal treatment approaches and to effectively manage changes in treatment needs over time.

The cost data drawn from four hospital settings, despite the caveats associated with the data, provides an indication of the impact that time-consuming and challenging diagnostic testing and personalisation of treatment can have on healthcare system resource demands, with costs concentrated in the early stages of the patient pathway. Further research is needed to understand healthcare service-related costs across a broader range of settings.

We have also identified key areas for future exploration, as part of a commitment to improving the diagnosis, treatment and management of patients with CDI and supporting patient quality of life and outcomes. Our research suggests a need to focus efforts on some key areas. We acknowledge that the extent to which these apply more widely—i.e. beyond the countries represented by the clinical co-authors (France, Ireland, Italy, Spain, and the UK)—merits further research. However, these insights are also supported by the challenges highlighted in the wider global literature.

More specifically, in reflecting on the challenges we have identified in this paper, there is scope to consider actions which could lead to improved guidance for primary care physicians on how to identify symptoms and manage patients with CDI. There is also a need for more information, education and awareness raising for patients, carers and families on how to better manage the condition, including in light of comorbidities and changes in patient circumstances. Working with patient associations may be important in this regard. Developing education and outreach for patients may also help patients engage with primary care and with specialists in secondary care.

Given the complexities of managing patients with CDI, further research is needed to understand whether there is potential for greater international consensus or guidance on best practice in the treatment and management of specific types of patients, not dismissing the importance of personalised care. This may require research that would gather retrospective and real-world data to identify how specific practices differ across a broad range of contexts and how they relate to patient outcomes for patients with specific clinical and behavioural parameters.

Finally, the COVID-19 pandemic introduced further challenges for patients with CDI, given high needs for inpatient care during diagnosis and treatment optimisation and there is scope for the clinical community to jointly consider how to approach patient care in the context of future pandemic preparedness.

We hope that the insights and reflections we have shared in this research help to raise awareness of the complexity of managing patients with CDI and support future efforts of clinicians, patient associations, policymakers and the community of patients and carers committed to improving the care of patients with this rare but life-impacting condition.

## Supplementary Information

**Additional file 1:** Methodology supplement.

## Data Availability

The data generated or analysed during this study are included in this published article and its Additional file 1. In line with informed consent, an exception is individual transcripts of interviews and workshop discussions due to risks of disclosing linkage of individual statements to individuals even when data is de-identified and a commitment to preserve anonymity. Further information outlining the methods used in this study is provided in the supporting information.

## Footnotes

1 Electronic Medicines Compendium. 2012. ‘DDVAP Melt 60mcg oral lyophilisate’. Emc. As of 22 December 2021: https://www.medicines.org.uk/emc/product/170/smpc#gref

2 OECD Data. 2021. ‘Purchasing power parities (PPP)’. OECD. As of 22 December 2021: https://data.oecd.org/conversion/purchasing-power-parities-ppp.htm#indicator-chart

3 Horizon Scanning Research & Intelligence Centre. 2016. ‘Low dose desmopressin lyophilisate (Noqdirna) for nocturia in adults. NIHR Innovation Observatory. As of 22 December 2021: https://www.io.nihr.ac.uk/wp-content/uploads/migrated/Low-dose-desmopressin-lyophilisate-Noqdirna-June16.pdf
