# Supplementary material for: Challenges and improvement needs in the care of patients with central diabetes insipidus
Source: Orphanet J Rare Dis. 2022 Feb 16;17:58. doi: 10.1186/s13023-022-02191-2 (PMC8848805; doi:10.1186/s13023-022-02191-2)
Supplement: Supplementary file 1 — Additional file 1: Methodology supplement. [file 13023_2022_2191_MOESM1_ESM.docx]

**Supplement 1: Methodology description**

**1 Narrative review methods**

26 papers were selected for analysis to provide an overview of the current state of knowledge in relation to the treatment and ongoing management of CDI. A subset of the papers also covers issues related to diagnosis.

The process for selecting 26 papers was as follows:

1. A search for relevant papers using the PubMed database and agreed search terms
2. Establishment of inclusion and exclusion criteria
3. A title and abstract screen of the longlist of retrieved papers to assess relevance
4. Prioritisation of papers to include in the review, in light of the inclusion criteria, relevance and scope of the study

A search was conducted, in October 2020, for papers published within the past 5 years on PubMed (2016-2020) using the search terms presented in Table 1, which cover different key permutations used to describe CDI and to capture papers with a particular interest in issues of treatment and management of patients with the condition. The initial search yielded 318 results, including reviews, case notes and clinical trials, books or book chapters. Using inclusion and exclusion criteria outlined in Table 1, an initial shortlist of 85 papers (62 yes, 23 maybe) was identified by two members of the research team through independent screening and subsequent discussion (HT and SM). As with any research study, relevant papers may have been published since the search was conducted, however these have not been included in our analysis as they did not fit within the study timeframe.

The research team was also interested in understanding medicines management and switching in light of the importance of personalised treatment of patients, and we conducted an additional search for papers on this topic using terms: (central diabetes insipidus) AND (switch *medic*) on PubMed, without filtering for year of publication due to the low volume of papers. We identified 4 relevant papers.

To prioritise papers within the scope of the research (given the balance of research focus on literature review and soliciting expert and patient representative views, time and resources), an initial list of 26 papers was prioritised as being of key interest and relevance for the focus of the research: i.e. on understanding challenges in the care of patients with CDI and areas in need of improvement. To arrive at this set of papers, HT and SM double screened a pilot of 65 papers, and subsequently the remainder of the papers were initially screened by HT. All papers deemed as relevant and papers for which there was uncertainty were also discussed by HT and SM to arrive at the final set. One of the shortlisted papers, on further analysis, was identified as a case report, which did not fit with the inclusion criteria, resulting in 25 papers in total.

Table 1. Search terms, inclusion and exclusion criteria

| Search terms and inclusion criteria | Exclude |
| --- | --- |
| **Articles (of any type apart from case notes) published in the period 2016-2020 focused on:**   - ‘central diabetes insipidus management’ or ‘central diabetes insipidus treatment’ - ‘neurogenic diabetes insipidus treatment’ or ‘neurogenic diabetes insipidus management’ - ‘neurohypophyseal diabetes insipidus treatment’ or ‘neurohypophyseal diabetes insipidus management’ - ‘vasopressin-sensitive diabetes insipidus treatment’ or ‘vasopressin-sensitive diabetes insipidus management’ - ‘central diabetes insipidus’ and switch *medic*^a^   **Where the paper provided significant detail on:**   - Treatment protocols, changing treatment needs, different modes of different modes of treatment delivery, sensitivity of treatment, ongoing monitoring and management of CDI, diagnosis of CDI.   **Where the paper provided an important overview of a specific cohort:**   - for example, adults, pregnancy, children and infants (although only where they clearly discuss treatment needs and management)   ^a^this search was not limited by date, due to the low number of papers it yielded. | **Articles published before 2016^b^**  **Articles where CDI was not the central focus of the paper, for example:**   - where the paper focused on other pituitary conditions or collections of diseases, such as Wolframs syndrome or Langerhans - related to traumatic brain injury, where CDI was referenced as a side-effect - where CDI was a transient result of post-operative surgery and the paper focused on other areas of care and recovery - Papers that focused on other primary polyuria-polydipsia conditions   **Animal studies**  **Papers that did not have an English version**  **Case notes, editorials and conference proceedings were excluded**  ^b^Aside from 2 papers identified as particularly important for the subject matter of medicines management for which there was less recent literature, from 2013 and 2015. Treatment and diagnosis have not changed dramatically in the past decade. |

Papers were analysed thematically with data coded into categories reflecting key insights related to diagnosis, treatment initiation and management of patients with CDI and information on associated challenges and improvement opportunities.

Coding categories included:

- Aim of the paper
- Article type
- Setting
- Geography
- Methods used
- The key topics covered, included diagnosis, management and ongoing treatment

Nearly half of the papers included in the sample provided an overview of the disease, mostly a review of the literature (n=13)[1,6,7,8,9,10,11,12,20,22,23,24,25] with one systematic review,[15] and one commentary.[4] Several papers described themselves as original research articles (n=4),[5,13,16,19] including six clinical studies.[2,3,14,17,18,21]

Although a detailed assessment of study quality or risk of bias was outside the scope of the review, analysis of articles took on board considerations related to clarity of aims, methods and study settings. While most articles clearly conveyed their aims and methods, four papers had limitations in clear reporting on methodology. Thirteen papers described CDI treatment and management in more general terms, without explicitly situating the findings in a clearly specified geographical context (i.e. especially when papers seemed to draw evidence from multiple geographies).

**2 Workshop methods**

Following analysis of the papers, we identified key themes in diagnosis, treatment and management of CDI for further discussion with the clinical co-authors, each representing a different health system. The topic of diagnosis was covered in papers on management of CDI. The co-authors represent key experts in the field assembled at the onset of the project as an expert panel.

Two workshops were held in November 2020. The first focused on the findings of the literature review, drawing on the clinicians’ clinical experience (i.e. experiential knowledge) to explore these ideas in more depth. The second workshop focused more specifically on the challenges to patient care and opportunities for improvement related to healthcare systems, and implications for overall findings from the research and contributions to the evidence base..

In preparation for the workshops, individual conversations were conducted between a RAND Europe health services researcher (either SM, HT, DRR, or JF) and each individual clinician, a total of five one-hour discussions.

The interviews were audio recorded for the purposes of note-taking, to identify key themes to be discussed within the workshop.

The key topics which were explored during the interviews were:

- Their professional role and speciality relating to supporting patients with CDI
- How a patient navigates their health country’s health system
- The diagnosis, treatment and management options for CDI in their country, and challenges experienced in the patient care pathway
- Significant areas of progress and improvement over time for diagnosis, treatment and management of CDI
- Focused discussion on:
  - Challenges affecting decision-making
  - Guidelines for patient support
  - Dose optimisation
  - Side-effects and management
  - Challenges for ongoing monitoring and management
  - Challenges related to the suitability of treatments for specific patient groups (including patients with comorbidities- i.e. other simultaneous conditions)
- Switching between different medicines and formulations
- The impact of COVID19 on diagnosis, management and treatment of CDI
- Their knowledge of best practice in other countries
- Insights into the cost of managing patients and what influences cost
- Wider healthcare system related influences on patient care

Detailed interview notes were produced by the research team from RAND Europe, and key themes identified though thematic analysis, which formed the basis for the workshops.

The workshops were each 90 mins in duration, supported by Microsoft teams.

The first workshop included a presentation of the literature review findings, and subsequent discussion with clinicians around their experiences of supporting patients with CDI. The second workshop focused on the health sector influences and shaping the different sections of the paper.

**3 Interviews with patient representatives of associations active in the CDI field**

To gather views from patient organisations, a search was conducted to find relevant organisations that supported patients with CDI in the countries of interest. This began with a search of the Orphanet database of organisations and selected by country, the search included pituitary foundation organisations, which may also have specific knowledge of CDI. Google searches of the conditions and the country were also conducted to find organisations directly. This yielded organisations in the UK, Italy, France and Ireland. The clinical co-authors of this research were also consulted for their awareness of relevant organisations, and this corroborated the findings from the search. Representatives from each organisation were identified through their website and contacted to request an interview. Positive responses were received from patient organisations in Ireland, UK and Italy.

Each patient representative was interviewed by either JR or GCA, using Microsoft teams. The interviews lasted approximately 45 mins and were recorded to aid notetaking and analysis, with participant consent. Patient Representatives were provided with details of the study in advance of the interview, along with a privacy notice, and completed an informed consent form before participating.

The interviews were semi-structured, following a topic guide which covered the themes of interest:

- Their role in the association
- How a patient with central diabetes insipidus navigates the healthcare system – the typical patient pathway including first contact, referrals, diagnosis, treatment decisions and ongoing management and monitoring
- The types of diagnosis options which exist in the country of interest
- The types of treatment options which exist in the country of interest and how are decisions about treatments, formulations and dosages are made
- Ongoing management and monitoring options for patients with CDI in the country of interest
- The key challenges related to diagnosing, treating and caring for patients with CDI in the country of interest
- Whether there has been any progress and improvement over time in terms of how patients are diagnosed, how they are treated, how they are managed and monitored
- Whether patients think it makes a difference whether they are prescribed branded or generic versions of CDI drugs
- Whether patients switch treatments over the course of their care (e.g. switch formulations, switch branded and generic treatments)
- Whether COVID-19 has had an impact on the diagnosis, treatment, and management and monitoring of patients with CDI
- Where there is opportunity for improvement of patient care for patients with CDI
- Whether there is a need for more patient education and awareness raising about the condition and how to manage it
- Whether there are any out of pocket costs/payments for patients associated with managing the condition
- Whether there are any other similar patient associations in other countries

The patient representative from Italy was known to have been involved in European Reference Networks, and therefore an additional question was included about her understanding of the broader European context for patients with CDI, and whether she had specific understanding of what was happening at a European level.

The interview notes were summarised and analysed for key themes, which were incorporated into the paper alongside the findings from the literature review and expert workshops.

Thematic analysis and synthesis of information was conducted to bring together insights form the literature review, workshops and focused discussion with clinical experts and interviews with patient representatives.
